# Atraumatic Splenic Rupture Unveiling Mumps With an Underlying B-cell Lymphoid Hyperplasia: A Diagnostic Conundrum

**DOI:** 10.7759/cureus.72671

**Published:** 2024-10-29

**Authors:** Inderjeet Singh, Hariharasudhan Balaji, Nithin Jyothy

**Affiliations:** 1 Internal Medicine, Southern Health and Social Care Trust, Craigavon, GBR; 2 Emergency Medicine, Southern Health and Social care trust, Craigavon, GBR; 3 Emergency Medicine, Southern Health and Social Care Trust, Craigavon, GBR

**Keywords:** atraumatic splenic rupture, hematological malignancy, ie splenic infarct, indications for surgery, mumps, splenic hematoma, total splenectomy

## Abstract

Atraumatic splenic rupture (ASR) is an unfamiliar entity that is potentially life-threatening if there is a delay in the diagnosis. Due to its rarity and its non-specific presentation, it can be a challenge to diagnose early. In this report, we present a case of a 42-year-old male patient who presented to the emergency department with nonspecific abdominal pain and had no past medical history. The patient presented abdominal pain associated with nausea, vomiting, and sweating. On examination, the patient was found to be tachycardic and mildly hypotensive, with mild left upper quadrant tenderness, and a lactate of 4 mmol/L on venous blood gas analysis. He was urgently transferred to the resuscitation area, where resuscitation commenced. Further investigations revealed significant anemia. The contrast-enhanced CT of the abdomen performed revealed a 13-cm splenic hemostasis suggestive of non-traumatic splenic rupture. The patient lacked any history of blunt trauma or family history that could account for the splenic rupture. The patient was taken to the theatre by the surgical team as he remained unstable. He received four units of blood in the theatre and underwent splenectomy due to the spleen being unsalvageable. Post-operatively, the patient was admitted to the high-dependency unit (HDU) for close monitoring. Histological examination of the splenic tissue revealed B-cell lymphoid hyperplasia and negative PCR for clonality. The patient was found to be IgG-positive for mumps and was not vaccinated for MMR. Surgeons believe it is the main cause of ASR, given that little literature available establishes the same. The case highlights the importance of consideration of ASR in patients presenting with unexplained abdominal pain and hemodynamic instability, even without evidence of trauma. Early imaging and operative intervention are lifesaving. The histologic findings indicate that there may be an associated hemopoietic disorder, and this case highlights the need for clinicians to consider splenic involvement in patients with mumps who present with abdominal pain or signs of hemodynamic instability.

## Introduction

Atraumatic splenic rupture (ASR) is an unfamiliar entity that is potentially life-threatening if there is a delay in the diagnosis. Due to its rarity and its non-specific presentation, it can be a challenge to diagnose early. Various causes have been established for ASR. One of the rare causes is the Mumps. Mumps, a viral infection caused by the paramyxovirus, is typically associated with parotid gland swelling and mild systemic symptoms [1]. While vaccination programs have significantly reduced its incidence in developed countries, sporadic outbreaks still occur, particularly in close-contact settings [2]. Complications of mumps are generally rare in adults, with orchitis being the most common extra-salivary manifestation in post-pubertal males.

Splenic involvement in mumps infection is exceedingly rare, with only a handful of cases reported in the medical literature. Splenic complications typically manifest as splenomegaly or, in even rarer instances, splenic infarction [3]. ASR, a potentially life-threatening condition, has been documented in various infectious diseases like cytomegalovirus (CMV) [4], Epstein-Barr virus (EBV), etc., but is exceptionally uncommon in mumps.

This case report presents a unique instance of ASR in an adult male with confirmed mumps infection and underlying B-cell pathology. Histological examination of the splenic tissue revealed B-cell lymphoid hyperplasia and negative PCR for clonality. The rarity of this complication underscores the importance of maintaining a high index of suspicion for atypical presentations of common viral infections and hematological conditions. Furthermore, this case highlights the need for clinicians to consider splenic involvement in patients with mumps who present with abdominal pain or signs of hemodynamic instability. By documenting this rare occurrence, we aim to contribute to the existing body of knowledge regarding mumps complications and to alert healthcare providers to the possibility of serious splenic involvement in what is often considered a benign childhood illness.

## Case presentation

A 42-year-old male presented to the emergency department with a four-day history of non-specific abdominal pain associated with nausea, vomiting, and sweating. The patient's medical history was unremarkable, with no prior significant illnesses or known immune conditions. His vaccination status was not explicitly stated in the provided information.

On initial examination, the patient was found to be tachycardic and mildly hypotensive. Physical examination revealed mild left upper quadrant tenderness. The patient denied any recent trauma or injury.

Initial venous blood gas analysis revealed an elevated lactate level of 4 mmol/L, indicating tissue hypoperfusion. Due to these concerning findings, the patient was urgently transferred to the resuscitation area where further resuscitative measures were initiated. Subsequent laboratory investigations revealed significant anemia, though specific hemoglobin levels were not provided.

**Figure 1:**
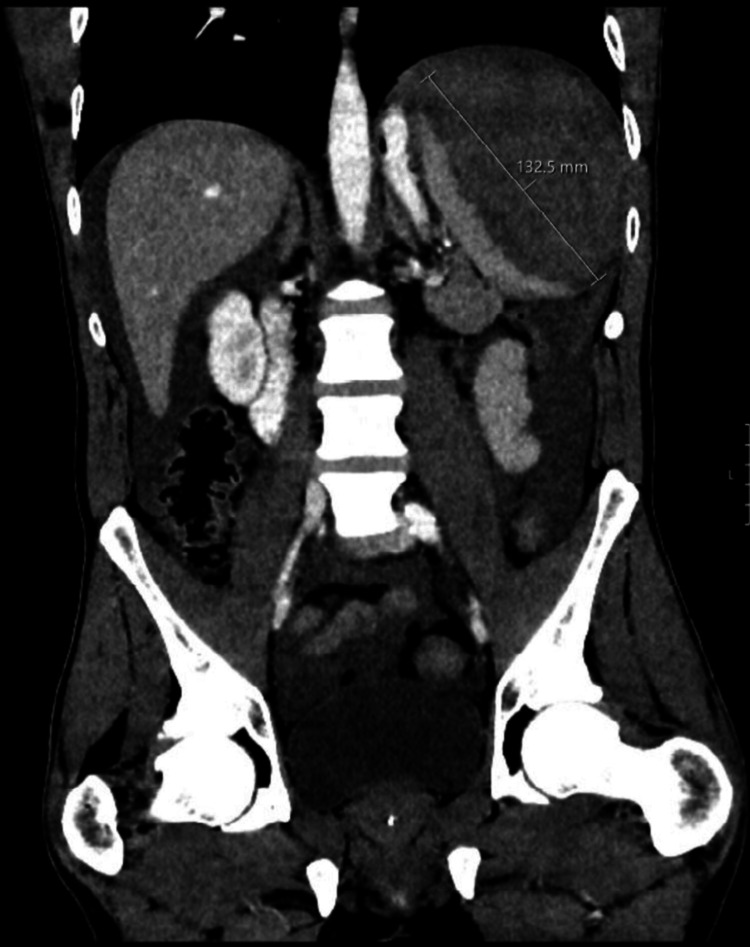
A contrast-enhanced computed tomography scan of the abdomen, revealing a 13.25cm splenic hematoma.

A contrast-enhanced computed tomography (CT) scan of the abdomen was performed (Figure 1), which revealed a 13.25 cm splenic hematoma, suggestive of non-traumatic splenic rupture. No other significant abdominal pathologies were noted on imaging. The diagnosis of ASR was made based on the clinical presentation of abdominal pain and hemodynamic instability, in conjunction with the CT findings of splenic rupture and the absence of any history of trauma.

Given the patient's unstable condition and the imaging findings, emergency surgical intervention was deemed necessary. The patient was taken to the operating theatre by the surgical team. Intra-operatively, a significant hemoperitoneum of 3.9 liters was encountered. Due to the extent of splenic damage, the organ was deemed unsalvageable, and a total splenectomy was performed. During the procedure, the patient received four units of blood to address the significant blood loss.

Postoperatively, the patient was admitted to the high-dependency unit (HDU) for close monitoring. The postoperative course was not detailed in the provided information, but it can be inferred that the patient's condition stabilized following the intervention.

**Figure 2:**
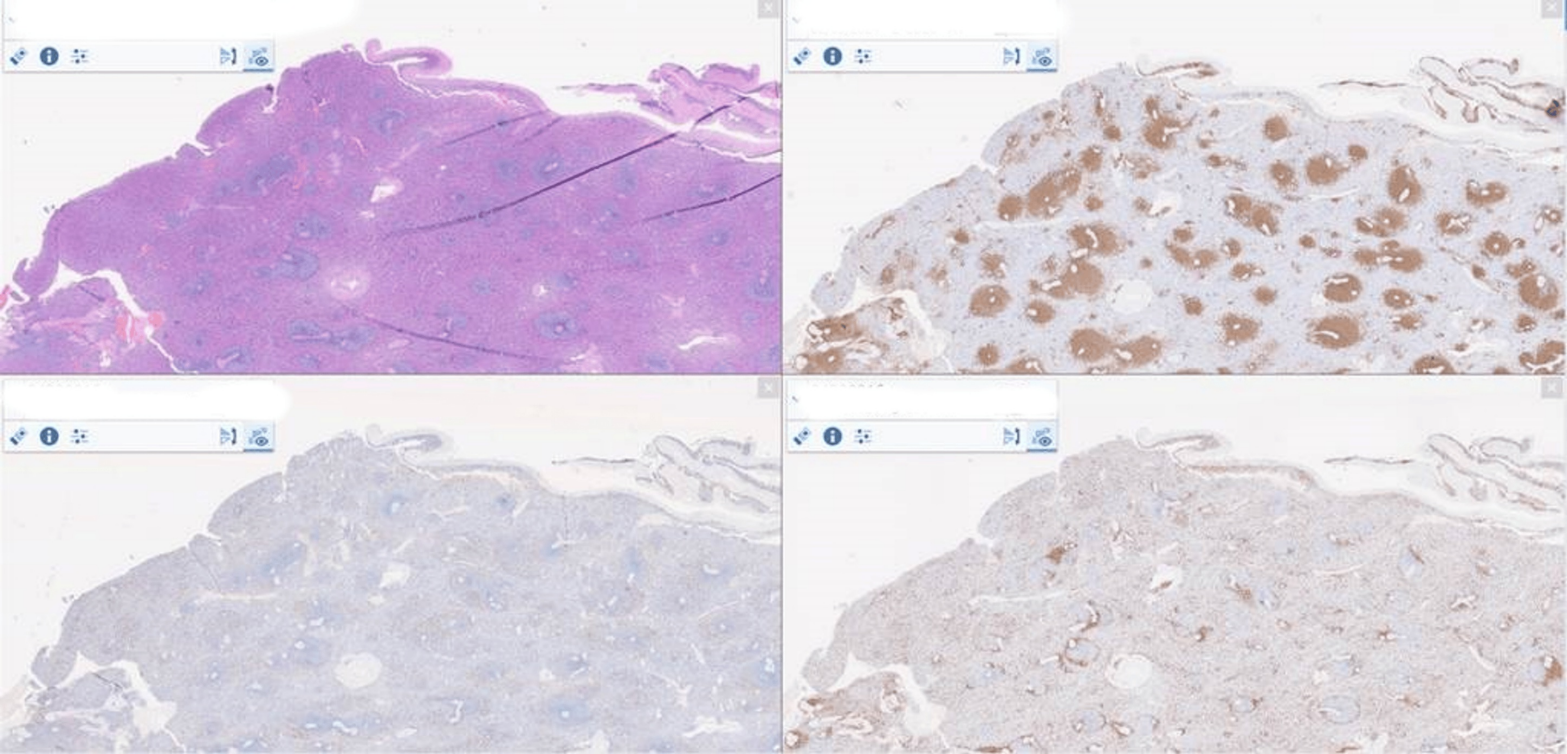
Histopathology of the splenic tissue revealed B-cell lymphoid hyperplasia.

Histological examination of the splenic tissue revealed B-cell lymphoid hyperplasia (Figure 2). Polymerase chain reaction (PCR) testing for clonality was negative. The patient was found to have a positive mumps IgG, suggesting the past infection as he was not vaccinated for MMR, established an important risk factor for splenic rupture. The surgeon believes this could be the cause of his ASR. Post surgery, the patient recovered well and was discharged home with the advice of the timely pneumococcal and influenza vaccination.

## Discussion

This brief case report describes an unusual occurrence where an adult male underwent ASR after contracting mumps. At first, the patient had nonspecific abdominal pain, tachycardia, and slight hypotension and thus there was no suspicion of an underlying serious condition. However, the rapid progression to hemodynamic instability and the subsequent discovery of significant hemoperitoneum underscore the importance of maintaining a high index of suspicion for splenic rupture, even in non-trauma settings.

This case underscores the importance of early identification and intervention in ASR. The initial atypical presentation may have been overlooked for prevalent abdominal conditions in most scenarios and it could have resulted in a delay in the diagnosis and treatment of ASR. Imaging the abdomen with a CT scan was essential for identifying the splenic rupture and facilitating immediate surgical intervention, and eventually proved to be lifesaving in this case.

ASR is a rare complication, with viral infections identified as an infrequent cause. Splenomegaly, advanced age, and neoplastic disorders are associated with increased ASR-related mortality [5]. Instances of ASR have been documented in connection with several viral infections, including EBV [6], CMV [7], and HIV; however, occurrences of mumps-related splenic rupture are notably infrequent in the literature. The impact of viral infections on lymphoid hyperplasia and spleen function is extensively documented, especially in the context of EBV and CMV infections. These viruses induce notable lymphocytic infiltration and proliferation in the spleen, resulting in splenomegaly and heightened fragility of the splenic capsule. The B cell lymphoid hyperplasia identified in the splenic tissue of our patient indicates a potential mechanism analogous to that seen in mumps, despite the latter's usual lack of severe splenic involvement [8].

It is essential to recognize that, although the patient exhibited signs of a previous mumps infection (positive IgG), a definitive causal relationship between mumps and splenic rupture cannot be established. Various mechanisms can enhance the risk of spleen rupture by viral infections. Therefore, viral-induced lymphoid hyperplasia results in fast enlargement of the spleen, which augments intrasplenic pressure and distends the splenic capsule. Even in the total absence of marked trauma, thus, increased spleen volume with potential variations in splenic architecture and enhanced tissue friability may make it more liable to ruptures in some cases [9].

On the other hand, particular viral infections can be associated with vasculitis or the formation of microthrombi within splenic vessels leading to areas of infarction that weaken its structural strength. Consequently, given the fact that there was an observed B-cell lymphoid hyperplasia in the splenic tissue, it may be due to a proliferative response hence causing an increase in the intrasplenic pressure which could result in its rupture.

## Conclusions

This case of ASR in an adult with past mumps infection highlights the importance of considering rare complications in seemingly benign viral illnesses. It underscores the need for clinicians to maintain a high index of suspicion for splenic rupture in patients presenting with abdominal pain and hemodynamic instability, even without trauma history. The non-specific initial presentation and rapid progression to life-threatening conditions emphasize the critical role of early recognition and intervention. Key recommendations include maintaining a low threshold for advanced imaging, particularly contrast-enhanced CT, in unexplained abdominal pain cases with hemodynamic changes. Additionally, a comprehensive evaluation of underlying hematological disorders is crucial in ASR cases. This report serves as a reminder of the potential for serious sequelae in common viral infections and the need for vigilance in the diagnosis and management of such rare complications.
